# Comparative 16S rRNA profiling of broiler chicken cecal microbiota following *Bacillus* lipopeptide or enrofloxacin administration

**DOI:** 10.1128/mra.01164-25

**Published:** 2026-01-20

**Authors:** Guzel Lutfullina, Daria Pudova, Marat Lutfullin, Yaw Akosah, Elena Shagimardanova, Margarita Sharipova, Ayslu Mardanova

**Affiliations:** 1 Department of Microbiology, Institute of Fundamental Medicine and Biology, Federal University, Kazan, Russia; 2 Laboratory of Agrobioengineering, Institute of Fundamental Medicine and Biology, Federal University, Kazan, Russia; 3 Department of Molecular Pathobiology, New York University College of Dentistry, New York, New York, USA; 4 Loginov Moscow Clinical Scientific Center, Moscow, Russia; California State University San Marcos, San Marcos, California, USA

**Keywords:** *Bacillus subtilis*, antimicrobial lipopeptides, 16S rRNA, chicken, sequencing, amplicon, antibiotic, enrofloxacin, microbiota of the cecum

## Abstract

We report 16S rRNA gene sequencing data characterizing the cecal microbiota of 35-day-old Ross 308 broiler chickens supplemented with a crude fraction of *Bacillus subtilis* GM5 lipopeptides in comparison with the fluoroquinolone antibiotic enrofloxacin. Microbial community analyses revealed distinct compositional shifts in response to peptide supplementation versus antibiotic treatment.

## ANNOUNCEMENT

The emergence of antibiotic-resistant microorganisms poses a growing challenge to poultry production and public health (1), highlighting the need for alternative strategies for microbial management . Antimicrobial peptides (AMPs) produced by *Bacillus* spp. are a promising class of biocontrol agents due to their potent, targeted antimicrobial activity, low likelihood of resistance development, and structural and functional diversity (2). Lipopeptides, a major class of AMPs, exhibit broad-spectrum antibacterial and antifungal activity and are under investigation as potential alternatives to conventional antibiotics in poultry (3).

We studied the cecal microbiota of broiler chickens in response to dietary supplementation with a crude lipopeptide fraction from *Bacillus subtilis* GM5 or the antibiotic enrofloxacin. A total of 90 1-day-old Ross 308 broiler chicks were randomly allocated to three groups: control (standard feed, *n* = 30), lipopeptide-supplemented feed (10 mg/kg, *n* = 30), and enrofloxacin-supplemented feed (50 mg/kg, *n* = 30). Treatments were administered from days 1 to 35 under controlled environmental conditions. All procedures adhered to the ethical standards of Kazan Federal University and Directive 2010/63/EU on the protection of animals used for scientific purposes.

On day 35, nine birds per group were randomly selected for cecal content collection. Following sequencing, seven samples were retained for the control group, five for the peptide group, and eight for the antibiotic group. Birds were euthanized by cervical dislocation, followed by decapitation, and cecal contents were collected aseptically. Immediately after euthanasia, the abdominal cavity was opened; both ceca were dissected; and their contents were placed into sterile 3 mL tubes, flash-frozen in liquid nitrogen, and stored at –80°C until DNA extraction (4). Genomic DNA was extracted from 500 mg of cecal content using the QIAamp Fast DNA Stool Mini Kit (QIAGEN, Germany) following the manufacturer’s instructions. DNA quality and concentration were assessed using a NanoDrop 2000 spectrophotometer (Thermo, USA) and agarose gel electrophoresis. The V3–V4 region of the 16S rRNA gene was amplified with primers 341F (5′-CCTACGGGNGGCWGCAG-3′) and 805R (5′-GACTACHVGGGTATCTAATCC-3′) (5), with multiplex identifiers and Q5 Hot Start High-Fidelity 2× Master Mix (NEB, UK) (6). Sequencing was performed on the Illumina MiSeq platform using a 250-bp paired-end library. Sequencing reads of each sample were processed using QIIME2 v2020.8 (7). Primer removal was performed with cutadapt, and denoising, paired-end merging, and chimera removal were conducted using the DADA2 plugin (8), generating a feature table of amplicon sequence variant (ASV) counts. ASVs were taxonomically classified with the Greengenes 16S rRNA database v13_8 (9) at 99% sequence similarity.

**Fig 1:**
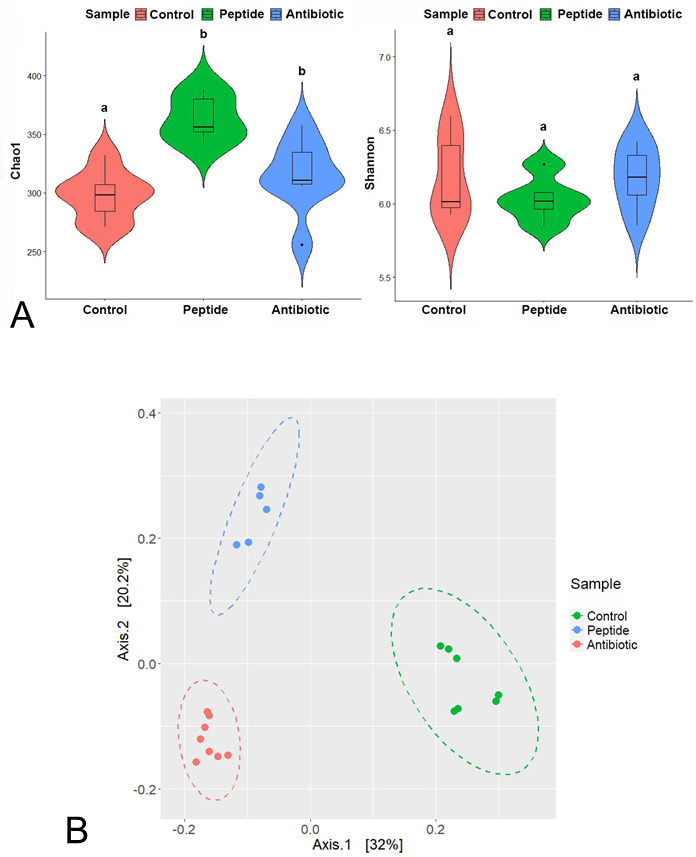
(**A**) Effect of dietary supplementation on *α*-diversity indices of cecal microbiota in broiler chickens at 35 days of age. Data are presented as mean ± SEM. Different letters (a, b) above the bars indicate a statistically significant difference (*P* < 0.05) between groups. Groups that share the same letter are not significantly different. (**B**) Principal coordinate analysis (PCoA) depicting differences in the cecal bacterial community structure of broiler chickens at 35 days of age in response to dietary treatment.

Across 20 samples, sequencing yielded 1,834,045 raw read pairs with an average of 61,695 ± 16,767 high-quality reads per sample (Table 1). In total, 1,233,908 reads were assigned to 934 bacterial ASVs spanning 17 phyla, 26 classes, 50 orders, 88 families, and 236 genera. *Firmicutes* dominated the control (59.5%) and enrofloxacin groups (39.5%), whereas *Bacteroidota* were most abundant in the lipopeptide group (45.7%). Alpha diversity (Shannon, Chao1 indices) and beta diversity (Bray–Curtis dissimilarity) were computed in R v4.3.3 (10) using phyloseq v1.46.0 (11), with statistical analyses conducted using vegan v2.6.4 (12) and ggplot2 v3.4.4 (13). One-way ANOVA, followed by Tukey’s HSD test, was used to assess group differences (*P* < 0.05). Lipopeptide supplementation significantly increased microbial richness (Fig. 1A), and beta diversity (Fig. 1B) analyses revealed distinct community structures between treatment groups, reflecting the differential effects of lipopeptides and antibiotics on the cecal microbiota.

**TABLE 1:** Summary of metadata and SRA accession numbers of the 16S RNA amplicon sequences of broiler cecal samples

Sample	Group	Number of reads	SRA accession number
C1	Control	55.9k	SRR34918367
C2	Control	80.1k	SRR34918366
C3	Control	54.3k	SRR34918355
C4	Control	70.9k	SRR34918354
C5	Control	70.1k	SRR34918353
C6	Control	96.7k	SRR34918352
C7	Control	87.2k	SRR34918351
P1	Peptide	94.4k	SRR34918350
P2	Peptide	87.1k	SRR34918349
P3	Peptide	104.6k	SRR34918348
P4	Peptide	134.4k	SRR34918365
P5	Peptide	131.4k	SRR34918364
A1	Antibiotic	94.9k	SRR34918363
A2	Antibiotic	91.9k	SRR34918362
A3	Antibiotic	113.7k	SRR34918361
A4	Antibiotic	128.0k	SRR34918360
A5	Antibiotic	88.4k	SRR34918359
A6	Antibiotic	89.8k	SRR34918358
A7	Antibiotic	95.8k	SRR34918357
A8	Antibiotic	64.3k	SRR34918356

The findings of this study serve as a potential foundation for developing novel applications of *Bacillus*-derived antimicrobial lipopeptides and for mitigating the toxic side effects of antibiotic use in poultry production.

## Data Availability

The raw 16S rRNA sequencing data are available at the NCBI Sequence Read Archive (SRA) under BioProject acccessionaccession number PRJNA1303408 with individual accession numbers (SRR34918348–SRR34918367), which are publicly available through this BioProject. The code used for analysis is available in the GitHub repository [https://github.com/dasha171711/broiler-microbiota-analysis]. The specific version of the code used for this manuscript is identified by the commit hash [335a77088885d470221bbd3020503669db4bf9c3].
